# The effect of being watched on facial EMG and autonomic activity in response to another individual’s facial expressions

**DOI:** 10.1038/s41598-019-51368-6

**Published:** 2019-10-14

**Authors:** Jari K. Hietanen, Anneli Kylliäinen, Mikko J. Peltola

**Affiliations:** Human Information Processing Laboratory, Faculty of Social Sciences/Psychology, FI-33014 Tampere University, Tampere, Finland

**Keywords:** Neurophysiology, Human behaviour

## Abstract

We tested if facial reactions to another person’s facial expressions depend on the self-relevance of the observed expressions. In the present study (n = 44), we measured facial electromyographic (zygomatic and corrugator) activity and autonomic arousal (skin conductance) responses to a live model’s smiling and neutral faces. In one condition, the participant and the model were able to see each other normally, whereas in the other condition, the participant was led to believe that the model could not see the participant. The results showed that the increment of zygomatic activity in response to smiling faces versus neutral faces was greater when the participants believed they were being watched than it was when the participants believed they were not being watched. However, zygomatic responses to smiles did not differ between the conditions, while the results suggested that the participants’ zygomatic responses to neutral faces seemed to attenuate in the condition of believing they were being watched. Autonomic responses to smiling faces were greater in the belief of being watched than in the belief of not being watched condition. The results suggest that the self-relevance of another individual’s facial expression modulates autonomic arousal responses and to a lesser extent facial EMG responses.

## Introduction

Other people’s facial expressions are crucial stimuli in our social environment. Facial expressions have been suggested to have two types of functions: they can signal the expressers’ social intentions and underlying emotions^1,2^. Interestingly, perceiving the facial expressions of other individuals can elicit facial reactions also in the observers, a phenomenon dubbed ‘facial mimicry’^3^. After Dimberg’s seminal findings^4,5^, numerous psychophysiological studies using facial electromyography (EMG) have shown that seeing another person’s facial expression can automatically elicit facial reactions on one’s own face that are congruent with the muscular activity producing the other individual’s facial expression (see^6^ for an extensive review). These facial reactions may reflect simple motor mimicry reactions based on the functioning of automatic perception-behaviour links^7^, or they may be emotional reactions triggered by interpreting and understanding the expresser’s facial cue as his/her emotion in context^6,8,9^.

One central question in the field has considered the automaticity of the facial reactions. Earlier studies provided support for the automaticity of these reactions. For example, the latency of these responses is short; the facial EMG responses to facial expressions occur as early as 300–400 ms after stimulus onset^10^. By using backward masking, it has been shown that happy and angry facial expressions elicit congruent facial reactions even when the facial stimuli do not reach the level of conscious perception^11^. In experiments requiring participants not to react with their facial muscles to facial expression stimuli, it has also been shown that participants cannot avoid producing congruent facial reactions to positive and negative facial expressions^12^. However, more recent studies have indicated that the facial reactions can be modulated by concurrent emotional processes or by factors related to the nature of the social relationship between the interactants (e.g. in-group membership, co-operation, or social status)^13–17^.

A potentially critical factor influencing facial reactions in response to another person’s facial expressions relates to the self-relevance of the expression. The gaze direction of the expresser – the person exhibiting the facial expression – provides an effective cue for self-relevance. A direct (self-directed) gaze is an ostensive signal indicating that the expresser’s accompanying verbal and/or nonverbal signals are sent specifically to the observer^18^. The self-relevance of perceived emotional expressions has been proposed to play a fundamental role in the elicitation of emotional reactions^19,20^. Indeed, studies have shown that facial EMG responses differentiate more clearly between the happy and angry expressions of an avatar when the avatar’s gaze is directed at the observer compared to when the gaze is averted^21^, and zygomatic and corrugator responses to happy and angry expressions, respectively, have been shown to be greater when human and avatar expressers have a direct gaze compared to an averted gaze^22,23^. In their ‘Simulation of smiles’ model (SIMS), Niedenthal and colleagues suggested that when perceiving another’s smile, the somatosensory system can simulate the embodied experience of how the perceived smile feels, and that eye contact, as a signal of social relevance, serves to trigger an embodied simulation of the perceived facial expression^24^. Eye contact has been suggested to increase not only facial mimicry, but other types of nonverbal mimicry as well, possibly due to the feeling of being monitored by another^25^.

In the present study, we investigated whether self-relevance modulates observers’ facial reactions to another person’s facial expressions. As described above, the expresser’s gaze direction is one way to manipulate the self-relevance of an expression. However, investigating the effect of self-relevance by manipulating the expresser’s gaze direction is not ideal. First, it is possible that the observed differences in the reactions to faces with a direct and an averted gaze do not reflect the effects of gaze direction on self-relevance, but rather the effects of gaze direction on the observer’s visual attention. The perception of another’s direct gaze is known to catch the viewer’s visual attention, whereas an averted gaze triggers shifts of attention away from the face to the gazed-at direction^26–29^. Thus, observers may allocate more attention to faces with a direct gaze than to faces with an averted gaze, and this may result in greater responses to the expressions of an individual with a direct gaze. Secondly, according to the *shared signal hypothesis*, both facial expression and gaze direction can signal an expresser’s motivational approach-avoidance tendencies, and when the behavioural intent of these two signals matches, the processing of facial expression is enhanced^30,31^. A smiling face (a stimulus signalling approach) with a direct gaze (also a stimulus signalling approach) is a stronger net signal of approach motivation compared to a smiling face with an averted gaze (a signal of avoidance). Therefore, facial reactions in response to a smiling face with a direct versus an averted gaze could differ because of the effects of gaze direction on facial expression processing.

To investigate more specifically the role played by the self-relevance of facial expressions in facial mimicry, we showed faces only with a direct gaze to the participants, but manipulated the participants’ belief that they were being watched by the person exhibiting the facial expressions. Recently, we showed that when participants were led to believe that a half-silvered mirror was placed between the participant and a live model in such a way that the model could not see the participant, psychophysiological responses (e.g. event-related brain potentials, heart rate, and autonomic arousal) and behavioural responses (e.g. reaction times in visual attention tasks) were not greater to the direct gaze than they were to the averted gaze, unlike when the half-silvered mirror was absent and the participants believed they could be seen by the model^32,33^. In the present study, we decided to use this same methodology to investigate what kind of role the experience of being watched by a live model – i.e. being the target of another’s attention – has on the participants’ facial EMG responses to the model’s affiliative smile and neutral face. We decided to focus on the affiliative smiles because an affiliative smile is probably the most typical facial expression we encounter in our daily lives^34,35^. In addition to facial EMG, we also measured autonomic arousal responses (skin conductance responses, SCR). Previous studies have shown that seeing a smiling face indeed elicits greater autonomic arousal than seeing a neutral face^36^; moreover, autonomic arousal responses to a live person’s smiling face are greater when the person is looking towards the observer as opposed to looking away^37^. These findings suggest that autonomic arousal is also sensitive to the self-relevance of another person’s smile.

To sum up, in the present study, we measured (i) the facial EMG responses from the muscle areas of the *Zygomaticus major* (cheek area, prototypical in happy facial expressions) and the *Corrugator supercilii* (brow area, prototypical in angry facial expressions and showing decreased activity during positive emotions) and (ii) the SCRs of participants who were facing a live model who looked directly at the participant and either smiled or had a neutral expression on his/her face. Importantly, using a procedure similar to our previous studies^32,33^, we collected data both in a condition where the participant and the model expressing the facial expressions were able to see each other and in a condition where the participants believed that the model could not see them. In both conditions, the visual input was identical. We wanted to investigate whether the participants’ facial reactions and autonomic arousal are influenced by their belief that they could be seen by the model. We expected that seeing another person’s affiliative smile would result in greater facial EMG and autonomic arousal responses compared to seeing another person with a neutral face. Critically, in line with models suggesting that self-relevance increases nonverbal mimicry to social stimuli^24,25^, we expected that these differences would be greater when the participants believed they were being watched by the model compared to when the participants believed they were not being watched by the model.

## Methods

### Participants

The participants (*N* = 44) were 22 females (mean age = 24.4, *SD* = 6.1) and 22 males (mean age = 26.0, *SD* = 6.5) recruited from undergraduate psychology courses. Apart from two males, all participants were right-handed and had normal or corrected-to-normal vision. The participants received course credits for their participation. The experimental protocol was approved by the ethics committee of the Tampere region. All participants gave their written, informed consent. The study conformed to the Code of Ethics of the World Medical Association (Declaration of Helsinki).

### Stimuli and procedure

The stimuli were a (static) smiling and a neutral face with a direct gaze posed by a live female or male model. In the smiling face condition, the models were trained to pose a natural, closed-mouth smile that they thought they could reliably reproduce from trial to trial. The smiles involved light muscle contractions in the eye region, but they were not full-blown enjoyment (or ‘Duchenne’) smiles with strong *Orbicularis oculi* activity^38^. For the neutral expression, the models were instructed to remain calm and expressionless. The facial expressions were presented through a 30 × 40 cm custom-built electronic shutter. The shutter was made of a voltage-sensitive liquid crystal (LC) window (NSG UMU Products Co., Ltd.) attached to a black frame. The model and the participant were sitting on opposite sides of the shutter. In order to minimize variability in the facial expressions, the models started to pose the expression before the electronic shutter was ‘opened’ (see below), and there were no dynamic changes (or other facial and head movements) in the expression during the stimulus presentation. The participant’s distance from the LC shutter was 70 cm and the model’s distance from the shutter was 40 cm. The model’s seat was adjusted so that the model’s and the participant’s eyes were at the same level vertically. E-Prime 2.0 software (Psychology Software Tools, Pittsburgh, PA) running on a desktop computer controlled the transparency (transparent or opaque) of the LC shutter. The shutter switched between opaque and transparent states in 3 milliseconds. The duration of each stimulus presentation trial was 5 s. The inter-stimulus interval (ISI) varied from 10 to 20 s. During the ISI, the shutter remained opaque. The duration of the ISI depended on how quickly the level of skin conductance returned to a pre-stimulus level. The opening of the shutter was controlled by an experimenter, who was sitting behind a movable partition 2 m behind the participant and monitoring the physiological signal online. Behind the partition, there was also a video camera focused on the LC shutter recording the model’s face throughout the experiment. Each participant saw only one model’s facial expressions. Half of the female and half of the male participants saw the female model and the other half saw the male model.

The stimuli were presented in two separate blocks. In one block, the participants were aware that the model was able to see them when the LC shutter was transparent (belief of being watched, BW). In the other condition (belief of not being watched, BnW), the participants were led to believe that the model’s vision was blocked. This belief was created by introducing the participant to a bogus half-silvered mirror. In reality, this was just a sheet of transparent plexiglass in a thin black frame. This plexiglass was attached to the LC window and slid between the participant and the model so that the participant saw an extra sheet being inserted in the LC window. The experimenter then took the participant to the other side of the shutter. Meanwhile, the model placed another sheet with an opaque surface in front of the LC window. When the participant arrived on the model’s side, he/she was able to notice that it was now impossible to see through the window. When the participant was taken back to his/her own side of the shutter, the model removed the opaque sheet unbeknownst to the participant (an illustration and a more detailed description of this procedure is presented in a previous study^33^). The order of the blocks (BW and BnW) was counterbalanced across the participants. Within a block, there were a total of 20 trials (10 with happy expressions and 10 with neutral expressions). The presentation order of the trials within a block was pseudorandom (no more than two consecutive trials had the same expression).

Upon arrival at the laboratory, the participants were told that the purpose of the study was to measure the perception of facial expressions during interaction with another person. The participants were then told that they would see one of the experimenters on the other side of the LC window posing different facial expressions. It was not mentioned at any point that there would be only happy and neutral expressions. To conceal the purpose of the experiment, the participants were told that their skin temperature would be measured during the experiment with electrodes attached to their face and fingertips. After the attachment of the electrodes, the participant and the model were seated in their places. The participants were instructed to sit and remain relatively motionless during the trials (i.e. when the model was visible) and to look towards the model. The participants were told that after each trial, they should use the keyboard in front of them to evaluate the model’s facial expression on a nine-point scale (1 = highly negative; 9 = highly positive).

Between the two stimulus presentation blocks, the participants filled short pen-and-paper questionnaires. First, the model opened the shutter window twice to show the participants the smiling or neutral face, with the order of the expressions counterbalanced across the participants. After both stimulus displays, the participant rated on a seven-point scale the model’s *approachability* (1 = very unapproachable; 7 = very approachable) and *dominance* (1 = very submissive; 7 = very dominant) as well as his/her own sentiments of *valence* (1 = pleasant; 7 = unpleasant) and *arousal* (1 = calm; 7 = tense) during the preceding trial. Finally, the participants filled the seven-point Social Presence Scale^39^ with one extra item (1 = ‘I felt very much socially present’; 7 = ‘I did not feel socially present at all’). The same questionnaires were administered again after the second stimulus block.

After the experiment, the participants were asked (both in writing and orally) about how they felt during the experiment and whether they felt differently between the situations in which the model could and could not see them. The purpose of this inquiry was to find out if the participant had any suspicion of deceit. During the debriefing, the participants were told about the deceit and they were asked if they had had any doubts about the model seeing them during the BnW belief block.

## Physiological Measures

### Facial muscle activity

Facial muscle activity over the *Zygomaticus major* and *Corrugator supercilii* was measured. The skin was cleaned with disinfectant. Bipolar 4 mm Ag-AgCl electrodes filled with electrode paste were attached 1 cm apart over the muscle sites according to the placement guidelines^40^. A ground electrode was attached to the middle of the forehead below the hairline. For the first 22 participants, the signal was continuously recorded with PowerLab amplifiers running LabChart software (http://www.adinstruments.com/) with a sampling rate of 2000 Hz, a 10–500 Hz bandpass filter, and a 50 Hz notch filter. For the remaining 22 participants, the signal was recorded with the same settings but using a QuickAmp amplifier running BrainVision Recorder software (Brain Products GmbH, Munich, Germany).

Offline, the EMG signal was visually inspected for artefacts caused by muscle movements and blinks, and 9.9% of the trials, on average, were excluded. Then, the signal was rectified, smoothed, and averaged from a 500 ms baseline (prior to stimulus onset) to 5000 ms post-stimulus in bins of 500 ms. These values were standardized for each participant in order to reduce the influence of extreme values. The final analyses were based on change scores. For these scores, the baseline muscle activity was subtracted from each 500 ms average value and then averaged across all accepted trials in each experimental condition.

### Skin conductance

Two electrodes (Ag/AgCl) filled with isotonic paste were attached to the palmar surface of the medial phalanxes of the index and middle fingers of the participant’s left hand. The signal was re-sampled offline to 100 Hz and filtered with a low-pass filter of 10 Hz. The response was defined as a maximum amplitude change from the baseline level (at the stimulus onset) during a time window of 4 s, which started 1 s after the stimulus onset. If the maximum amplitude was below 0.01 µS or negative, it was set to zero. If the amplitude rise during the first second after stimulus onset (before the 4-s response window) was more than 0.1 μS, the trial was rejected. Due to technical problems, SCR data from 10 participants were rejected. For the remaining 34 participants, 14.8% of all trials were eliminated due to the above-mentioned rejection criterion. The data (including zero responses) were averaged in each condition for each participant. This calculation combines response size and response frequency and results in the *magnitude* of the skin conductance response^41^.

### Post-experiment evaluation of the video recordings of the models’ expressions

As noted above, the participants were asked to evaluate the negativity−positivity of each facial expression after each trial. These data were collected in order to check whether the participants experienced any difference in the intensity of the smiles between the BW and BnW blocks. However, because the participants were told that the model would be watching them in one condition, it was possible that this knowledge could have biased the participants’ evaluations somehow.

Therefore, we decided to use the video recording of the stimulus faces and ask a new sample of participants to evaluate the expressions from these recordings. From all the video recordings during the data collection from 40 participants (the video recordings of four participants were contaminated due to experimenter error – e.g. the video camera was not switched on), we randomly selected one trial from each condition (Expression × State of belief, i.e. 4 different stimuli) per participant and edited them to video clips lasting 6.5 s. Thus, we had a set of 40 video clips from each stimulus condition. Half of these featured the female model and the other half featured the male model. From these video clips, we edited the following stimulus sequence. In each trial, a fixation point (3000 ms) was presented against a white background. After this, the video clip was started. During the first second of the video clip, the shutter remained opaque. It then turned transparent for 5 s showing the stimulus face before turning opaque again for 0.5 s. After this, a display was presented asking the participant to evaluate the model’s facial expression on a nine-point scale (1 = highly negative; 9 = highly positive). These sequences contained expressions from both the female and the male model. The stimuli were presented in four blocks, with each block presenting facial expressions from one model recorded in one State of belief condition.

These stimuli were presented to 31 participants (21 females; mean age = 17.7, *SD* = 3.0) on a computer screen. As noted, these participants were not aware of the State of belief manipulation. They were simply asked to watch the stimuli and evaluate the facial expressions.

### Data analysis

All statistical analyses were conducted using repeated measures ANOVA. Planned comparisons were performed for the analysis of simple main effects when interactions were observed. A Greenhouse-Geisser correction procedure was applied when appropriate.

For the analyses of the EMG and skin conductance responses, the sex of the model and participant were originally included in the analyses. However, as the results showed that these factors had no main effects and did not interact with Expression or State of belief, they were not included in the final analyses reported in the results section below. Nevertheless, we present the results of the full analyses (i.e. including the sex of the model and participant) for the psychophysiological and the self-reported data (facial expression ratings and questionnaires) in the Supplementary Information. Although the order of presentation of the BW and BnW blocks was counterbalanced across the participants, we also conducted exploratory analyses on the main results by including block order as an additional independent variable.

## Results

### Facial expression rating

**Figure 1:**
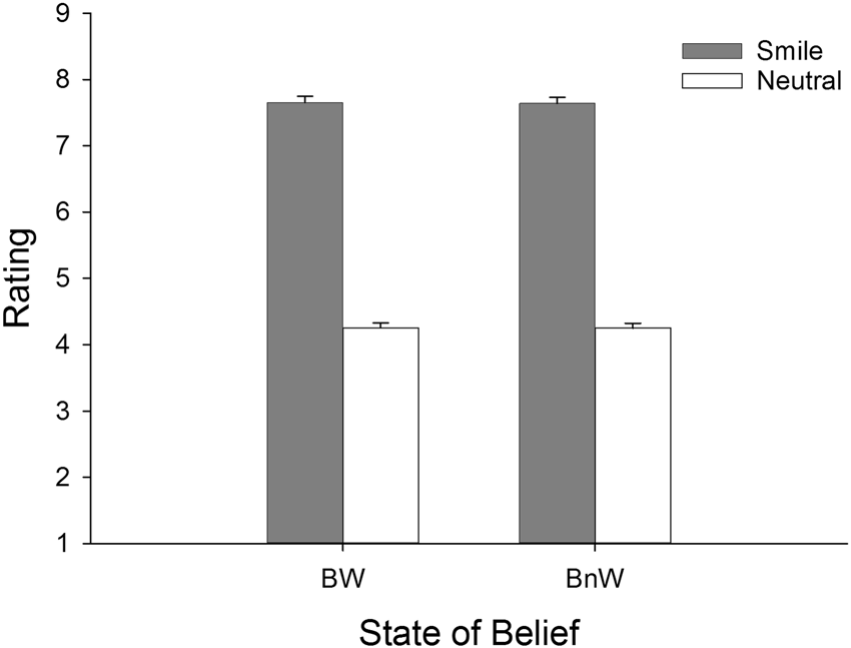
Means (and standard error of mean, SEM) of the ratings of the stimulus faces after each trial on a nine-point scale (1 = highly negative; 9 = highly positive) as a function of Expression and the State of belief.

The results of the participants’ facial expression ratings after each trial are shown in Fig. 1. A 2(Expression) × 2(State of belief) ANOVA showed that smiling faces (*M* = 7.64, *SEM* = 0.09) were rated as looking more positive than neutral faces (*M* = 4.24, *SEM* = 0.07; *F*(1,20) = 1058.39, *p* < 0.001, ${\eta }_{p}^{2}=0.96$). There was no main effect of State of belief (*p* > 0.81) nor interaction between the main effects (*p* > 0.90).

The ratings collected from the separate sample of participants looking at the video recordings of the stimulus expressions were also analysed with a 2(Expression) × 2(State of belief) ANOVA. This analysis showed that smiling faces (*M* = 6.86, *SEM* = 0.15) were rated as looking more positive than neutral faces (*M* = 4.16, *SEM* = 0.13; *F*(1,30) = 121.23, *p* < 0.001, ${\eta }_{p}^{2}=0.80$). Again, there was no main effect of State of belief, and most importantly, there was no interaction between the main effects (*p* > 0.1).

### EMG responses

**Figure 2:**
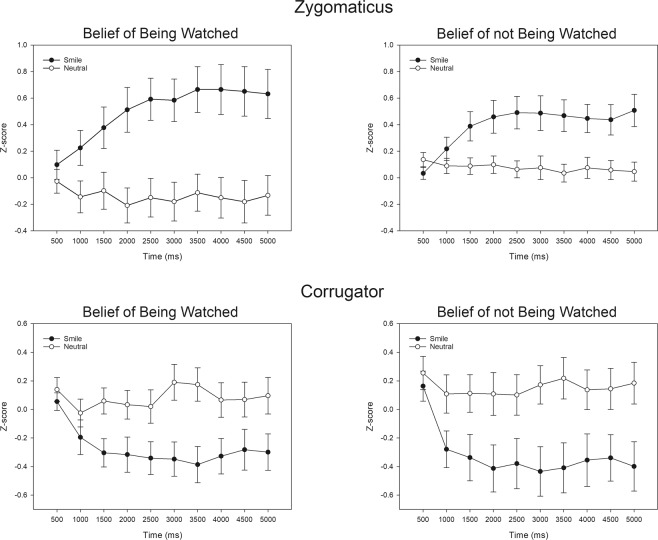
Mean zygomatic and corrugator electromyography (EMG) activity as a function of Expression, Time, and State of belief.

EMG responses in the zygomatic region were analysed with a 2(Expression) × 2(State of belief) × 10(Time) ANOVA (see Fig. 2). For zygomatic region responses, the ANOVA indicated significant main effects for Expression (*F*(1,43) = 17.76, *p* = 0.001, ${\eta }_{p}^{2}=0.29$) and Time (*F*(9,387) = 3.65, *p* = 0.017, ${\eta }_{p}^{2}$ = 0.08). The zygomatic muscle region responses were greater in response to smiling (*M* = 0.447, *SEM* = 0.101) than to neutral (*M* = −0.031, *SEM* = 0.076) faces. There was also an interaction between Expression × Time (*F*(9,387) = 11.86, *p* = 0.001, ${\eta }_{p}^{2}=0.22$), reflecting the fact that the increase of the zygomatic region activity as a function of Time was greater in response to smiling faces than to neutral faces. Most importantly, there was a significant interaction between Expression × State of belief (*F*(1,43) = 4.78, *p* = 0.034, ${\eta }_{p}^{2}=0.10$). Pairwise comparisons indicated that the zygomatic responses were greater for smiling faces compared to neutral faces in both the BW condition (*M*_*smile*_ = 0.500 vs *M*_*neutral*_ = −0.139, *p* = 0.001) and the BnW condition (*M*_*smile*_ = 0.394 vs *M*_*neutral*_ = 0.077, *p* = 0.012), but as indicated by the interaction effect, the difference between smiling and neutral faces was significantly greater in the BW block than in the BnW block. However, comparisons between the blocks did not clearly indicate whether the interaction was mainly driven by differential responses to smiling or neutral faces between the BW and BnW blocks. The comparisons were not significant in either the smiling (p = 0.448) or the neutral (p = 0.094) condition. The other interactions were not significant.

For corrugator region EMG responses, the analysis showed significant main effects for Expression (*F*(1,43) = 13.74, *p* = 0.001, ${\eta }_{p}^{2}=0.24$) and Time (*F*(9,387) = 5.81, *p* = 0.002, ${\eta }_{p}^{2}$ = 0.12). The corrugator muscle region responses decreased more in response to smiling faces (*M* = −0.296, *SEM* = 0.110) than to neutral faces (*M* = 0.118, *SEM* = 0.098). There was also an interaction between Expression and Time (*F*(9,387) = 7.92, *p* = 0.001, ${\eta }_{p}^{2}$ = 0.16), reflecting the fact that, as a function of Time, corrugator activity increased in response to neutral faces but decreased in response to smiling faces. The interaction between Expression and State of belief was not significant (*p* = 0.426).

### Skin conductance responses

**Figure 3:**
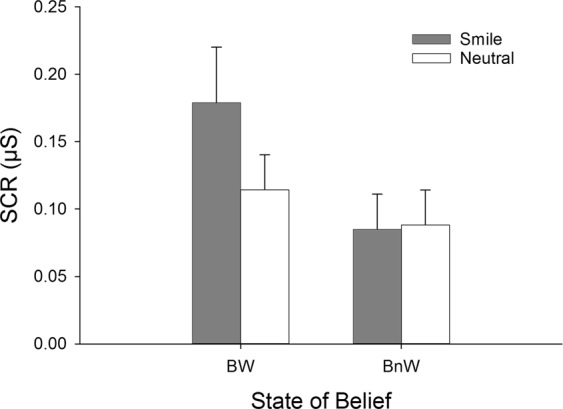
Mean skin conductance responses (SCR and SEM) as a function of Expression and State of belief.

Figure 3 shows the mean SCR values in response to both facial expressions in the BW and BnW conditions. The SCRs (ln-transformed values) were subjected to a 2(Expression) × 2(State of belief) analysis of variance (ANOVA). The ANOVA indicated significant main effects of Expression (*F*(1,33) = 5.98, *p* = 0.020, ${\eta }_{p}^{2}=0.15$) and State of belief (*F*(1,33) = 5.13, *p* = 0.030, ${\eta }_{p}^{2}=0.14$). Importantly the interaction between the main effects of Expression and State of belief was significant (*F*(1,33) = 9.44, *p* = 0.004, ${\eta }_{p}^{2}=0.22$). Pairwise comparisons indicated that the SCR was greater for smiling faces (*M* = 0.179, *SEM* = 0.041) compared to neutral faces (*M* = 0.114, *SEM* = 0.026) in the BW condition (*p* = 0.002), but not in the BnW condition (*M*_*smile*_ = 0.085, *SEM* = 0.026 vs *M*_*neutral*_ = 0.088, *SEM* = 0.026, *p* = 0.791). Comparisons between the blocks indicated that the SCR to smiling faces was significantly greater in the BW block than in the BnW block (*p* = 0.006), whereas the SCR to neutral faces did not differ between the BW and BnW conditions (*p* = 0.262).

### Questionnaires

As described in the methods section, the participants filled short pen-and-paper questionnaires after both stimulus presentation blocks. For the approachability, dominance, affective valence, and affective arousal ratings, the data were analysed with 2(Expression) × 2(State of belief) ANOVAs. For approachability, there was a main effect of Expression (*F*(1,43) = 436.05, *p* = 0.0001, ${\eta }_{p}^{2}=0.91$). Smiling faces were evaluated to be more approachable than the neutral faces (*M* = 5.97 vs *M* = 2.83). There was also a main effect of Expression for dominance ratings (*F*(1,43) = 4.53, *p* = 0.039, ${\eta }_{p}^{2}$ = 0.10). Neutral faces were evaluated to be more dominant compared to the happy faces (*M* = 4.28 vs *M* = 3.78). For feelings of affective valence, smiling faces were rated to elicit more pleasant feelings compared to the neutral faces (*M* = 5.56 vs *M* = 3.05; *F*(1,43) = 184.31, *p* = 0.0001, ${\eta }_{p}^{2}=0.81$). For subjective feelings of affective arousal, neutral faces were rated to be more arousing compared to smiling faces (*M* = 2.83 vs *M* = 2.25; *F*(1,43) = 7.39, *p* = 0.009, ${\eta }_{p}^{2}=0.15$). The main effect of State of belief and the interaction between the main effects were not significant for any of these ratings (*p* > 0.1)

Finally, the ratings from the social presence scale were analysed with a *t*-test. The results showed that the feelings of social presence were higher in the BW condition than in the BnW condition (*M* = 4.78 vs *M* = 4.12; *t*(43) = 4.77, *p* < 0.001).

### The effect of block order

As mentioned in the methods section, although the order of BW and BnW presentation was counterbalanced across participants, we also analysed whether the presentation order (i.e. BW–BnW vs BnW–BW) would have an effect on the main results. Therefore, we conducted 2(Expression) × 2(State of belief) × 2(Block order) ANOVAs for the behavioural expression ratings, psychophysiological data, and questionnaire measurements. For the EMG analyses, we omitted the factor Time in order to increase the power of the analyses.

For the participants’ ratings of the models’ facial expressions, the block order did not have a main effect (*p* = 0.944) nor did it interact with any of the other effects (all *p*-values > 0.330).

For the zygomatic region responses, the ANOVA indicated a significant main effect of block order (*F*(1,42) = 5.52, *p* = 0.024, ${\eta }_{p}^{2}=0.116$). The zygomatic responses were overall greater when the presentation sequence of the blocks was BnW–BW (*M* = 0.363) as opposed to BW–BnW (*M* = 0.052). Moreover, there was an Expression × State of belief x Order three-way interaction (*F*(1,42) = 5.05, *p* = 0.030, ${\eta }_{p}^{2}=0.107$). For the BW–BnW sequence, there was an Expression × State of belief interaction (*F*(1,21) = 9.87, *p* = 0.005, ${\eta }_{p}^{2}=0.320$). Pairwise comparisons indicated that in the BW condition, the zygomatic response was greater to smiling faces compared to neutral faces (*M*_*smile*_ = 0.326 vs *M*_*neutral*_ = −0.401, *p* = 0.004), but the difference in the BnW condition was not significant (*M*_*smile*_ = 0.187 vs *M*_*neutral*_ = 0.097, *p* = 0.579). Comparisons between the blocks indicated that zygomatic responses to smiling faces were not significantly different between the BW and BnW conditions (*p* = 0.577), whereas zygomatic responses to neutral faces were significantly smaller in the BW condition than in the BnW condition (*p* = 0.028). Notably, the zygomaticus was actually relaxed in the BW condition (in comparison to the pre-stimulus activity) in response to seeing another’s neutral face. For the BnW–BW sequence, the Expression × State of belief interaction was not significant (*p* = 0.977). The main effect of Expression was significant (*F*(1,21) = 12.08, *p* = 0.002, ${\eta }_{p}^{2}=0.365$), indicating that independent of the State of belief condition, zygomatic responses were greater to smiling faces compared to neutral faces (*M*_*smile*_ = 0.637 vs *M*_*neutral*_ = 0.090). For the corrugator region EMG responses, the block order did not have a main effect (*p* = 0.574) nor was there a significant interaction with any of the other effects (*p* > 0.08).

The analysis conducted on the SCR data indicated a significant State of belief x Block order interaction (*F*(1,32) = 14.44, *p* = 0.001, ${\eta }_{p}^{2}=0.311$). The interaction reflected that the autonomic responses were overall greater in the BW (*M* = 0.146) than in the BnW (*M* = 0.028) condition when the blocks were presented in the sequence BW–BnW, whereas there was no overall difference between the blocks when they were presented in the sequence BnW–BW (*M*_*BW*_ = 0.102 vs *M*_*BnW*_ = 0.122). Importantly, in both block order sequences, the SCR was greater for smiling faces compared to neutral faces in the BW condition, but not in the BnW condition. Other interactions involving block order were not significant (*p* > 0.17).

For the questionnaire data, the block order did not interact (all *p*-values > 0.10) with Expression or State of Belief for any of the measurements (i.e. approachability, dominance, affective valence, affective arousal, and feelings of social presence).

## Discussion

In the present study, we measured the participants’ facial EMG and autonomic arousal responses to another, live person’s smiling or neutral face. Our aim was to investigate whether these responses are influenced by the participant’s belief of being watched by the model expressing the facial expressions. To this end, the measurements were performed in two conditions. In one condition, the participant and the model were able to see each other, whereas in the other condition the participant was led to believe (by using a deception procedure) that the model was not able to see the participant. We expected that facial mimicry and autonomic arousal in response to a smiling versus a neutral face would be greater when the participants believed that the model was able to watch them, i.e. in a condition when the model’s facial expression was likely to be experienced as more self-relevant.

The results replicated numerous findings by showing that, in general, zygomatic EMG responses were greater and corrugator EMG responses were smaller to smiling faces compared to neutral faces^4,10,11,15,42^. More interestingly, however, the zygomatic muscle response differentiated more clearly between a smiling face and a neutral face when the participants believed they were seen by the model as compared to when they thought the model could not see them. This result is compatible with previous findings showing that facial EMG responses differentiate more clearly between perceived expressions^21^, and they are greater to expressions^22,23^ when associated with the expressers’ direct gaze compared to their averted gaze.

Rather unexpectedly, however, the additional exploratory analyses showed a significant effect of the presentation order of the State of belief manipulation blocks on the zygomatic responses. For participants with the presentation sequence BW–BnW (belief of being watched – belief of not being watched), the zygomatic responses were greater to smiling faces than to neutral faces only in the BW condition. In the BnW condition, the facial expression had no effect on the zygomatic responses. Instead, for participants with the presentation sequence BnW–BW, the zygomatic responses were greater to smiling faces than to neutral faces in both conditions and there was no effect of the State of belief manipulation. It is possible that this result reflects the automaticity of facial mimicry in response to smiles. When the experiment was started with the BnW condition and when the participants saw another person’s smiling or neutral face through the LC window for the first times, it is possible that the belief of the model’s inability to see through the window did not hinder the (natural) zygomatic reaction in response to another person’s smile. Instead, when the BnW block came second, the participants were able to contrast this with the experience of seeing another person in the BW condition, and the belief of not being watched manipulation was more effective. This explanation is compatible with results from the autonomic arousal measurements. The SCRs were overall greater in the BW condition than in the BnW condition only when the BW block came first, whereas there was no difference between the blocks when the BW block came second. Importantly, the observed significant effect of the block order indicates that even these types of contextual factors – perhaps especially when experimenting with relatively natural social interaction conditions – may have a considerable impact on the psychophysiological responses.

Although the results provided evidence that the zygomatic activity differentiated more clearly between a smile and a neutral face when the participants had a belief of being watched by the model as compared to when this was not the case, the present results did not show that zygomatic responses to smiles would have been greater in the BW block than in the BnW block. Instead, the present findings rather provide evidence for the automaticity of facial mimicry in response to the smiles of others. It has been proposed that in the course of human evolution, mimicry of others’ nonverbal behaviour has had a survival value via fostering relationships with others and, over time, nonverbal mimicry has become highly automatized, occurring without conscious awareness or intention and functioning as a ‘social glue’ between people^43,44^. The present results are compatible with these views. Another person’s smile triggered a smiling response, independent of whether the participants believed they were being watched.

A novel and somewhat unexpected finding is that zygomatic responses to neutral faces appeared to be sensitive to the State of belief manipulation. Analyses of the participants treated with the BW–BnW block sequence clearly showed that while there was no difference in the zygomatic responses to smiling faces between the BW and BnW blocks, the zygomatic response to neutral faces was significantly smaller in the BW block compared to the BnW block. In fact, the zygomatic muscle was relaxed (in comparison to the pre-stimulus level) in response to a neutral face in the BW block. Decreased zygomatic activity in response to neutral faces has been observed also in some previous studies^45^. Thus, the present results suggest that the participants may have a heightened awareness of not smiling in response to another’s neutral face when they know to be watched by the other person. Inhibition of a smile in response to seeing another person’s neutral face may be related to ‘saving one’s face’ – i.e. withdrawing from sending an affiliative signal in a situation when such a signal is not received from an interaction partner. It is also very likely that in these types of experiments, facial responses to a given facial expression depend on the context of other presented facial expressions. In the present study, neutral faces alternated with smiling faces, and it is possible that in this context, neutral faces are perceived more negatively than they would be, say, in the context of angry or sad faces.

Compared to the zygomatic responses, the effect of the State of belief manipulation was more robust on the autonomic arousal responses, and the presentation order of the experimental blocks did not have an influence on the Expression x State of belief interaction. Seeing the model’s affiliative smile compared to a neutral face resulted in greater SCRs only in the BW condition independent of whether this block was presented before or after the BnW block. In the BnW condition, the smile had no effect on participants’ autonomic arousal. This finding is also compatible with previous studies conducted with a live model as a stimulus showing increased autonomic arousal responses to smiling faces^36,37^. Moreover, the present result is in line with a previous finding that SCRs to a live model’s smiling face are greater when the model is looking towards the participant as compared to when looking away^37^. Interestingly, in many previous studies measuring autonomic responses to photographic facial expressions, facial expressions have had no or only marginal effects on skin conductance^46–50^. There are studies, for example, which have compared SCRs in response to facial expressions and affective pictures from the International Affective Picture System (IAPS^51^), and while SCRs to affective natural scenes were found, no effects of facial expressions were observed^49,50^. After their findings, Surcinelli and Codispoti^49^ concluded ‘The perception of emotional facial expressions does not seem to trigger the motivational system in the laboratory; however, these stimuli evoke facial reactions, possibly reflecting a mimicking behaviour which is of great interest to social sciences’ (p. S108). The present results are very much in accordance with this view.

The facial expression ratings after each trial expectedly indicated that smiling faces were rated as looking more positive than neutral faces. Critically, however, there was no difference in the ratings between the BW condition and the BnW condition. The order of the block presentation sequence also did not have an effect on these ratings. Furthermore, the post-experimental ratings of the video recordings with a new sample of participants showed that smiling faces looked more positive than neutral faces without any difference in these ratings between the BW and BnW conditions. These findings indicate that there was no difference in the intensity of the output expressions between the conditions. The results from the social presence measurements indicated that the feelings of social presence were higher in the BW condition compared to the BnW condition. This finding, together with the observed pattern of results from the physiological recordings, indicates that the manipulation worked as intended. In supplementary analyses, we also analysed the effects of the sex of the model and the participant on the psychophysiological and explicit rating responses (see Supplementary Information). The psychophysiological responses were not influenced by the sex of either the model or the participant. For explicit ratings, the results showed that female participants rated smiling male faces more positively than smiling female faces, while there was no effect of the model’s sex on the ratings of neutral faces. For male participants, there was no effect of the model’s sex on the ratings. Previous research has shown somewhat inconsistent results regarding whether gender affects facial responses to emotional expressions, but researchers generally agree that gender effects should be investigated more systematically in this research area, especially using naturalistic social stimuli^52^.

A limitation of the present study is that we investigated facial mimicry and autonomic arousal only in response to smiling and neutral faces. It remains to be determined whether the present results would also apply to other expressions. It is possible that because an affiliative smile is the most typical facial expression we encounter in our daily lives^34^, the facial responses have become more automatized in response to affiliative smiles compared to other expressions. Thus, it is possible that self-relevance could have a stronger effect on the mimicry of other facial expressions. This is obviously a question awaiting for empirical testing.

The present study showed that facial EMG responses to seeing a (live) person expressing an affiliative smile or a neutral face depended differently on the participants’ perception of being watched by the model. Although the difference in zygomatic responses to affiliative smiles versus neutral faces was diminished when the participants believed that they were not being watched by the model, additional analyses indicated that another person’s affiliative smile appeared to trigger a zygomatic response independent of whether the participants believed they were being watched or not, whereas the zygomatic response to neutral faces seemed to attenuate when the participants believed they were being watched. An autonomic arousal response to affiliative smiles versus neutral faces was found to be greater only when the participants believed that they were being watched. In sum, the present study resulted in two interesting and novel findings. First, the results provide evidence for the automaticity of facial mimicry in response to others’ smiles; another person’s affiliative smile triggers a smiling response, independent of whether the observer believes he/she is being watched or not. Second, the results suggest that awareness of being watched by another person may attenuate zygomatic responses in response to a neutral face.

## Supplementary information

Supplementary Information

## Data Availability

The datasets generated and/or analysed during the current study are available from the corresponding author on reasonable request.
