# Supplementary material for: The effect of being watched on facial EMG and autonomic activity in response to another individual’s facial expressions
Source: Sci Rep. 2019 Oct 14;9:14759. doi: 10.1038/s41598-019-51368-6 (PMC6791861; doi:10.1038/s41598-019-51368-6)
Supplement: Supplementary file 1 — Supplementary Information [file 41598_2019_51368_MOESM1_ESM.pdf]

SUPPLEMENTARY INFORMATION

The effect of being watched on facial EMG and autonomic activity in response to another individual's facial expressions

Jari K. Hietanen*, Anneli Kylliäinen, and Mikko J. Peltola

Tampere University

Author Note

Jari K. Hietanen, Anneli Kylliäinen, and Mikko J. Peltola, Human Information Processing Laboratory, Faculty of Social Sciences/Psychology, FI-33014 Tampere University, Finland.

Address for correspondence: Jari Hietanen, Human Information Processing Laboratory, Faculty of Social Sciences/Psychology, FIN-33014 Tampere University, Finland, e-mail: jari.hietanen@tuni.fi, phone: +358 40 190 1384.

Additional analyses and results

As described in the main text, for the analyses of the EMG and skin conductance responses, the model's sex and the participant's sex were originally included into the analyses. However, as the results showed that these factors had no main effects nor were they interacting with Expression or State of belief, these factors were not included into the final analyses reported in main text. In the following, we nevertheless present all these original results with both the model's sex and the participant's sex included as independent factors.

Zygomaticus and corrugator responses

The EMG responses were first analyzed with a 2(Expression) × 2(State of belief) x 2(Model's sex) x 2 (Participant's sex) ANOVAs. For these analyses, we omitted the factor Time in order to increase the power of these analyses.

For the zygomatic responses, only the main effect of Expression, $F(1,40) = 16.58$, $p < .001$, $\eta_p^2 = 0.29$, and the Expression x State of Belief interaction, $F(1,40) = 4.82$, $p = .034$, $\eta_p^2 = 0.11$, were significant. All the main effects or interactions involving Model's sex and/or Participant's sex were not significant (all *p*s > .1). The magnitude of the zygomatic responses as a function of Expression, State of belief, Model's sex, and Participant's sex are presented in Table S1. For the corrugator responses, only the main effect of Expression was significant, $F(1,40) = 13.77$, $p = .001$, $\eta_p^2 = 0.26$. All the main effects or interactions involving Model's sex and/or Participant's sex were not significant (all *p*s > .1). The magnitude of the corrugator responses as a function of Expression, State of belief, Model's sex, and Participant's sex are presented in Table S2.

Table S1. The magnitude of the zygomatic responses (mean and standard error of the mean) as a function of State of belief (BW vs. BnW), Expression (Smile vs. Neutral), Model's sex (F vs. M), and Participant's sex (Female vs. Male).

	BW				BnW			
	Smile		Neutral		Smile		Neutral	
Participant sex	F	M	F	M	F	M	F	M
Female	0.573	0.656	-0.164	-0.160	0.381	0.511	0.118	-0.044
	(0.308)	(0.282)	(0.267)	(0.244)	(0.200)	(0.183)	(0.123)	(0.113)
Male	0.640	0.072	0.170	-0.458	0.463	0.182	0.149	0.092
	(0.282)	(0.308)	(0.244)	(0.267)	(0.183)	(0.200)	(0.113)	(0.123)

Table S2. The magnitude of the corrugator responses (mean and standard error of the mean) as a function of State of belief (BW vs. BnW), Expression (Smile vs. Neutral), Model's sex (F vs. M), and Participant's sex (Female vs. Male).

	BW				BnW			
	Smile		Neutral		Smile		Neutral	
Participant sex	F	M	F	M	F	M	F	M
Female	0.094	-0.363	0.423	-0.230	-0.244	-0.352	0.289	-0.045
	(0.211)	(0.193)	(0.200)	(0.183)	(0.329)	(0.301)	(0.279)	(0.255)
Male	-0.212	-0.611	0.014	0.198	-0.274	-0.405	0.344	0.029
	(0.193)	(0.211)	(0.183)	(0.200)	(0.301)	(0.329)	(0.255)	(0.279)

Skin conductance responses

For the SCRs, the main effect of Expression, $F(1,30) = 6.70$, $p = .015$, $\eta_p^2 = 0.18$, the main effect of State of belief, $F(1,30) = 4.61$, $p = .040$, $\eta_p^2 = 0.13$, and the Expression x State of belief interaction, $F(1,30) = 8.76$, $p = .006$, $\eta_p^2 = 0.23$, were significant. All the main effects or interactions involving Model's sex and/or Participant's sex were not significant (all *p*s > .1). The magnitude of the SCRs as a function of Expression, State of belief, Model's sex, and Participant's sex are presented in Table S3.

Table S3. The magnitude of the skin conductance responses (mean and standard error of the mean) as a function of State of belief (BW vs. BnW), Expression (Smile vs. Neutral), Model's sex (F vs. M), and Participant's sex (Female vs. Male).

	BW				BnW			
	Smile		Neutral		Smile		Neutral	
Participant sex	F	M	F	M	F	M	F	M
Female	0.121	0.205	0.050	0.106	0.038	0.086	0.032	0.069
	(0.086)	(0.092)	(0.052)	(0.056)	(0.055)	(0.059)	(0.054)	(0.057)
Male	0.136	0.246	0.111	0.174	0.074	0.132	0.074	0.159
	(0.081)	(0.077)	(0.049)	(0.047)	(0.052)	(0.049)	(0.050)	(0.048)

Facial expression ratings

The data were analysed with a 2(Expression) × 2(State of belief) x 2(Model's sex) x 2 (Participant's sex) ANOVA. This analysis showed the main effect of Expression, $F(1,40) = 1199.57$, $p < .001$, $\eta_p^2 = 0.96$; smiling faces ($M = 7.64$) were rated to look more positive than the neutral faces ($M = 4.24$). Also, the Expression × Model's sex × Participant's sex interaction was statistically significant, $F(1,40) = 7.83$, $p < .01$, $\eta_p^2 = 0.16$. For female participants, the Expression × Model's sex interaction was significant, $F(1,20) = 8.74$, $p < .01$, $\eta_p^2 = 0.30$, reflecting that smiling male faces were rated more positively than smiling female faces ($M_{male} = 7.96$ vs. $M_{female} = 7.34$), while there was no effect of model's sex on the ratings of neutral faces ($M_{male} = 4.34$ vs. $M_{female} = 4.44$). For male participants, there was no effect of model's sex on the ratings. The mean ratings (and standard error of mean) as a function of Expression, State of belief, Model's sex, and Participant's sex are presented in Table S4.

Table S4. The facial expression ratings (positivity) as a function of State of belief (BW vs. BnW), Expression (Smile vs. Neutral), Model's sex (F vs. M), and Participant's sex (Female vs. Male).

	BW				BnW			
	Smile		Neutral		Smile		Neutral	
Participant sex	F	M	F	M	F	M	F	M
Female	7.320	7.975	4.470	4.308	7.350	7.942	4.400	4.375
	(0.190)	(0.173)	(0.154)	(0.141)	(0.188)	(0.172)	(0.151)	(0.138)
Male	7.633	7.620	3.983	4.270	7.633	7.570	3.925	4.310
	(0.173)	(0.190)	(0.141)	(0.154)	(0.172)	(0.188)	(0.138)	(0.151)

Questionnaires

There was only a main effect of expression for approachability, $F(1,40) = 414.98$, $p = .0001$, $\eta_p^2 = 0.91$, for affective valence, $F(1,40) = 173.18$, $p = .0001$, $\eta_p^2 = 0.81$, and a marginal effect for dominance ratings, $F(1,40) = 3.94$, $p = .054$, $\eta_p^2 = 0.09$. None of the other main effects or interaction effects were significant (*p*s > .1). For subjective feelings of affective arousal, the main effects of expression, $F(1,40) = 6.85$, $p = .012$, $\eta_p^2 = 0.15$, and model's sex, $F(1,40) = 4.50$, $p = .04$, $\eta_p^2 = 0.10$, were significant. Female faces were overall rated more arousing than male faces ($M = 2.82$ vs. $M = 2.23$). Other effects were not significant. For the ratings of the social presence, the main effect of State of belief, $F(1,40) = 21.70$, $p = .0001$, $\eta_p^2 = 0.35$, was the only statistically significant effect.
